# Supplementary material for: Distribution and Risk Factors of Malaria in the Greater Accra Region in Ghana
Source: Int J Environ Res Public Health. 2022 Sep 22;19(19):12006. doi: 10.3390/ijerph191912006 (PMC9566805; doi:10.3390/ijerph191912006)
Supplement: Supplementary file 1 [file ijerph-19-12006-s001.zip › ijerph-1896699-supplementary.pdf]

Supplementary Materials

Supplementary Table S1: Poisson and Negative Binomial (Full Model)

**Model**	**AIC**
Poisson	187082.88
Negative Binomial*	25123.72

* selected due to lower AIC

AIC- Akaike Information Criterion

Supplementary Table S2: Variance Inflation Factor (VIF)

**Covariate**	**VIF**	**1/VIF**
Rainfall	1.49	0.669487
Maximum Temperature	3.29	0.303940
Minimum Temperature	3.30	0.303083
Infections (log base 1.1)	1.19	0.838308
Population density (log base 1.1)	1.17	0.853458

VIF- variance inflation factor

Supplementary Table S3: Comparing model with and without minimum and maximum temperature.

**Models**	**Variables**	**AIC**
Model 1*	Rainfall<br>Tmin**<br>Malaria cases of previous month log transformed<br>Population Density	23354.09
Model 2	Rainfall<br>Tmax***<br>Malaria cases of previous month<br>Population Density	23379

* Model 1 selected due to lower AIC; ** minimum temperature; *** maximum temperature

AIC- Akaike Information Criterion

Supplementary Table S4: Summary table of reported cases and climatic variables by district

	**Total Reported Cases**	**Projected Population (2019 GSS)**	**Population Density**	**Annual Parasite Index (API)**	**Minimum Temp (°C)**	**Maximum Temp (°C)**	**Monthly rainfall (mm)**
Adenta Municipal District	69758	95077	1377.9	146.74	23.90	32.06	83.20
Ledzokuku Municipal District	39394	168707	5442.2	46.70	23.98	31.89	78.83
Ada East District	32175	87820	336.5	73.27	24.66	32.36	86.68
Shai-Osudoku District	48989	63499	75.4	154.30	24.01	32.43	93.40

Ada West District	23941	72653	254	65.91	24.40	32.41	89.88
Ningo-Prampram District	66714	87248	161.9	152.93	24.02	32.34	88.68
La-Dade-Kotopon Municipal District	17437	226047	7064	15.43	24.06	32.02	76.66
La-Nkwantanang-Madina Municipal District	51889	137706	2295.1	75.36	23.65	31.79	86.32
Ga East Municipal District	68924	183032	2731.8	75.31	23.39	31.42	88.08
Ayawaso West Municipal District	1739	92997.67	2999.9	3.74	23.92	31.95	79.93
Ga South Municipal District	57602	338042.1	1310.2	34.08	23.53	31.54	96.17
Ga West Municipal District	54642	151039	686.5	72.35	23.66	31.62	92.00
Ga Central Municipal District	36113	144058	3065.1	50.14	23.80	31.50	86.53
Tema West Municipal District	6369	141384.1	3141.9	9.01	24.05	32.22	82.38
Ashaiman Municipal District	187322	236123	13889.6	158.66	23.94	32.18	83.78
Kpone-Katamanso Municipal District	93987	134852	648.3	139.39	23.72	31.92	86.44
Ablekuma Central Municipal District	3355	212786.9	23643	3.15	24.10	32.18	79.32
Korle-Klottey Municipal District	20227	162497.2	16249.7	24.90	24.06	32.02	78.00
Ablekuma North Municipal District	11356	191075.5	17370.5	11.89	24.01	31.83	81.60
Ayawaso North Municipal District	10142	96517.88	48258.9	21.02	23.92	31.95	79.93
Ayawaso East Municipal District	5981	128247.9	42749.3	9.33	23.912	31.95	79.93
Okaikwei North Municipal District	21544	254746.9	13407.7	16.91	24.08	32.25	81.42
Ga North Municipal District	49129	123578	2686.5	79.51	23.27	31.27	92.10
Weija-Gbawe Municipal District	25038	194305.9	4134.2	25.77	23.79	31.29	83.98
Krowor Municipal District	6241	112472	7029.5	11.10	23.95	31.86	77.56
Tema Metropolitan District	16993	219288.9	6449.7	15.50	24.01	32.09	78.51
Ablekuma West Municipal District	13122	203524.3	20352.4	12.89	23.96	31.61	75.76
Ayawaso Central Municipal District	7523	246226.2	41037.7	6.11	23.92	31.95	79.93
Accra Metropolitan District	57724	550212.7	27510.6	20.98	24.10	32.18	79.32

Supplementary Table S5: Rainfall and case numbers in the Greater Accra Region by month

	Jan	Feb	Mar	Apr	May	Jun	Jul	Aug	Sep	Oct	Nov	Dec
Rainfall (mm)	9.41	35.1	87.3	88.1	147.3	215.7	61.4	39.4	96.0	117.8	79.3	27.3
Mean malaria incidence	18323.2	16175.4	18399	16950.4	17304.2	21628	22416.6	19516.2	16477.8	18677.8	18856.8	16348.6
Maximum Temperature	33.09	34.13	33.65	33.48	32.43	30.38	29.29	28.95	30.02	31.67	32.95	33.17
Minimum Temperature	23.16	24.76	24.52	24.84	24.51	23.95	23.26	22.90	23.32	23.78	24.21	23.86

Supplementary Figure S1 Global Moran's I

Moran's Index: 0.111008

z-score: 3.379896

p-value: 0.000725

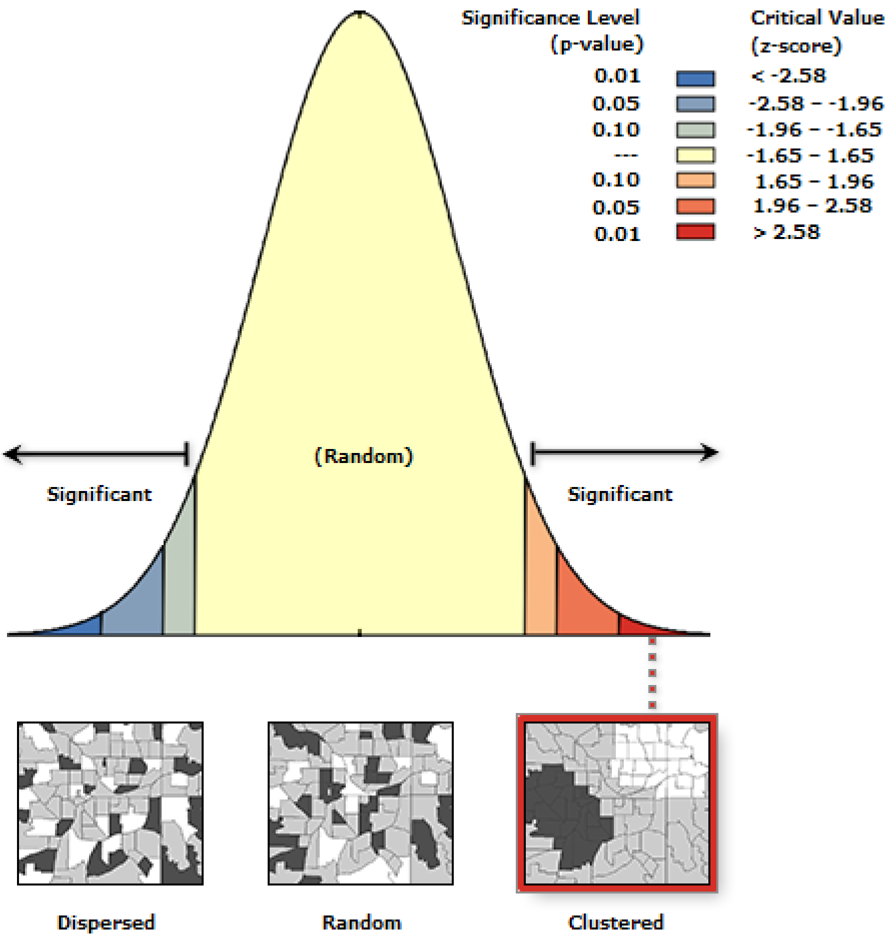

Given the z-score of 3.37989648414, there is a less than 1% likelihood that this clustered pattern could be the result of random chance.

Global Moran's I Summary

**Moran's Index:**	0.111008
**Expected Index:**	-0.035714
**Variance:**	0.001884
**z-score:**	3.379896
**p-value:**	0.000725

Dataset Information

**Input Feature Class:**	Data_for_global_morans
**Input Field:**	INF
**Conceptualization:**	FIXED_DISTANCE
**Distance Method:**	EUCLIDEAN
**Row Standardization:**	False
**Distance Threshold:**	17035.2662 Meters
**Weights Matrix File:**	None
**Selection Set:**	False
